# Harnessing the synergistic potential of biologically targeted therapies in cutaneous T-cell lymphoma

**DOI:** 10.18632/oncotarget.26751

**Published:** 2019-03-08

**Authors:** Christina Chung Patrone, Larisa J. Geskin

**Affiliations:** Memorial Sloan Kettering Cancer Center, New York, NY, USA; Associate Professor of Dermatology and Director of the Comprehensive Skin Cancer Center, Department of Dermatology, Columbia University Irving Medical Center, New York, NY, USA

**Keywords:** CTCL, cutaneous T cell lymphoma, romidepsin, histone deacetylase, BET inhibitor

**Background**

Oncology research is currently blossoming with biologically targeted cancer therapies as alternatives to traditional chemotherapies. Cutaneous T-cell lymphoma (CTCL) in particular has been historically challenging to treat in advanced stages, as conventional cytotoxic regimens are poorly tolerated and have limited long-term efficacy. Newer immunological agents and small molecule inhibitors have quickly become essential tools in the CTCL armamentarium, however response rates remain limited and relapses continue to occur.

**The need for combination therapy**

Mutational analysis has revealed that CTCL is quite genetically heterogeneous, but certain trends can be observed, including mutual genetic exclusivity [1], thus the disease requires a multi-faceted therapeutic approach. Recent in vitro studies have examined various combination therapies that could act synergistically in CTCL to ultimately boost response rates. Histone deacetylase (HDAC) inhibitors romidepsin and vorinostat, currently FDA-approved as a monotherapy for CTCL, have been popular choices for combination in vitro experiments and have yielded promising results. HDAC inhibitors create epigenetic modifications that pair well with other targeted and cytotoxic therapies to further alter gene expression in CTCL. For example, combinations of HDAC inhibitors with nitrogen mustard (mechlorethamine) [2], lenalidomide [3], proteasome inhibitor bortezomib [4], and BCL2 inhibitor venetoclax [5], have all shown success in vitro with more than additive effects.

**Using genomics to identify viable targets**

The use of whole genome sequencing, whole exome sequencing, and gene expression profiling has aided some of these studies to target specific somatic mutations or copy number variants in CTCL. Sequencing aids in selecting logical combinations and elucidating the mechanisms through which these combinations are effective. The NFκB pathway is constitutively activated in CTCL and downregulation of this pathway leads to apoptosis in CTCL [6]. A key player within the NFκB pathway of therapeutic interest is the *MYC* oncogene, which is often amplified in CTCL [7]. Expression of *MYC* can be repressed by bromodomain and extraterminal (BET) protein inhibitors. Kim et al. examines the use of targeting *MYC* using four different small-molecule BET inhibitors in vitro on CTCL patient samples and cell lines: JQ1, which has a short-half life, and I-BET762, CPI- 0610, and ABBV-075, which have more favorable pharmacological characteristics [8]. JQ1 and ABBV-075 were the two most effective BET-inhibitors for reducing cell viability in CTCL samples as single agents. IC50s were in the micromolar range, with up to 10-fold variation. The reason for this variability was unclear, as MYC copy number did not correlate to sensitivity in samples with a copy number alteration.

JQ1 and ABBV-075 were then tested in combination with HDAC inhibitors vorinostat and romidepsin and BCL2 inhibitor venetoclax, and found to work synergistically with these agents in patient samples. BET inhibitors combined HDAC inhibitors or BCL2 inhibitors demonstrated increased apoptosis than single agents alone. Results among 9 patient samples were more consistent when BET inhibitors were combined with HDAC inhibitors than with BCL2 inhibitors. BET inhibition and HDAC inhibition together significantly reduced MYC and BLC2 gene expression.

**Discussion**

We recently published a pooled genomic analysis from seven deep sequencing studies with 139 CTCL cases [1], the largest genomic analysis of raw sequencing data to date. Using this dataset, we confirmed the importance of TP53 and NFκB pathway derangements previously identified in CTCL. We identified mutual exclusivity between cases harboring TP53 pathway mutations and NFκB/KIT pathway mutations. Somatic copy number variation did not follow this pattern, and cases with both *TP53* deletion and *MYC* amplification were seen. *TP53* deletion leads to upregulation of BCL2 [5]. Combining a BET inhibitor with an HDAC inhibitor hits both crucial pathways: the NFκB pathway via *MYC* and the TP53 pathway via *BLC2*, thereby providing a logical approach to combination therapy for CTCL.

Ultimately, the combination therapies with the best clinical utility will be the ones that succeed. For example, we have shown that mechlorethamine and romidepsin exhibit synergy in vitro in CTCL and gene expression profiling using RNAseq revealed downregulation of the JAK-STAT pathway, another key dysregulated pathway in CTCL [2]. As these are two already FDA-approved therapies for CTCL, studying them clinically is quite feasible. BET inhibitors are in early stages of development. JQ1 is impractical for clinical use due to its short half-life. Among the other BET inhibitors studied, ABBV-075 showed the most potent effects against CTCL in vitro. ABBV-075 (Mivebresib) is currently being studied in phase 1 clinical trials in solid tumors and AML, with preliminary data showing efficacy and tolerability [9]. Further translational and clinical studies could provide head-to-head comparisons of eligible candidate agents for the best marriage with HDAC inhibitors.
